# Supplementary material for: Molecular insights into ago-allosteric modulation of the human glucagon-like peptide-1 receptor
Source: Nat Commun. 2021 Jun 18;12:3763. doi: 10.1038/s41467-021-24058-z (PMC8213797; doi:10.1038/s41467-021-24058-z)
Supplement: Supplementary file 1 — Supplementary Information [file 41467_2021_24058_MOESM1_ESM.pdf]

Supplementary Information

Molecular insights into ago-allosteric modulation of the human glucagon-like peptide-1 receptor

Brief description of what this file includes:

Supplementary Fig. 1 Functional validation of the receptor constructs and purification of the complexes.

Supplementary Fig. 2 Cryo-EM data processing and validation.

Supplementary Fig. 3 Near-atomic resolution model of the complexes in the cryo-EM density maps.

Supplementary Fig. 4 Comparison of available GLP-1R structures.

Supplementary Fig. 5 Unique ECD conformation in the compound 2-bound GLP-1R.

Supplementary Fig. 6 Comparison of G protein coupling between compound 2-bound and GLP-1-bound active GLP-1R in complex with G_s.

Supplementary Fig. 7 Molecular dynamics (MD) simulations of compound 2-bound active GLP-1R.

Supplementary Fig. 8 Potentiation of GLP-1 and LY3502970 activity by compound 2.

Supplementary Fig. 9 MD simulations of compound 2–GLP-1–GLP-1R.

Supplementary Fig. 10 List of small molecule GLP-1R modulators with available structures.

Supplementary Table. 1 Cryo-EM data collection, refinement and validation statistics.

Supplementary Table. 2 *In vitro* pharmacology of GLP-1R.

Supplementary Table. 3 *In vitro* pharmacology of GCGR.

Supplementary Table. 4 Effects of residue mutation in the ECD-ECL1 interface on cAMP accumulation.

Supplementary Table. 5 Details of restraints applied during MD simulations.

Supplementary Table. 6 Primers used in this study.

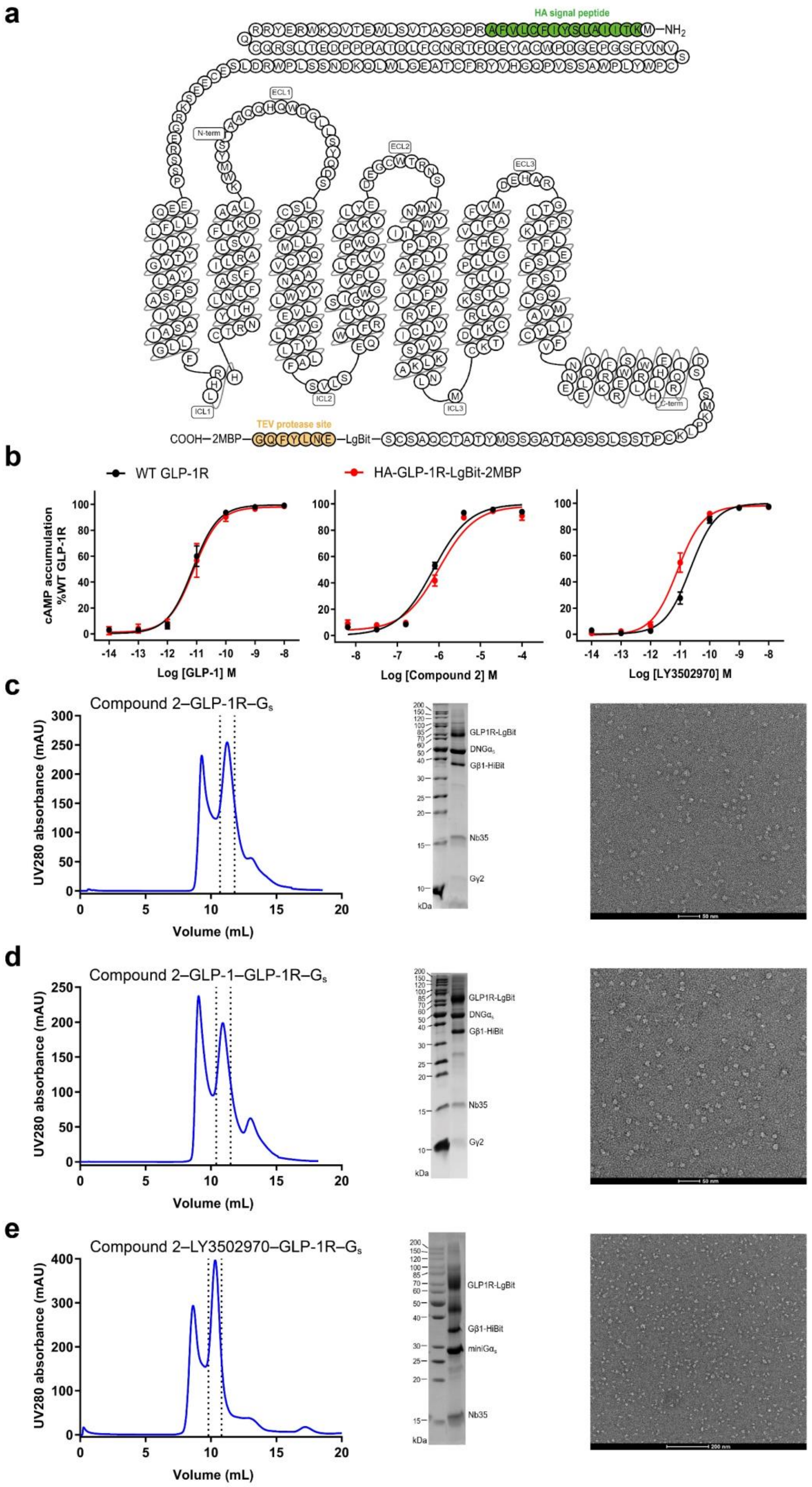
a
HA signal peptide
TEV protease site
COOH–2MBP–
–LgBit–
–NH2
ECL1
ECL2
ECL3
ICL1
ICL2
ICL3
N-term
C-term
b
WT GLP-1R
HA-GLP-1R-LgBit-2MBP
cAMP accumulation %WT GLP-1R
Log [GLP-1] M
Log [Compound 2] M
Log [LY3502970] M
c
Compound 2–GLP-1R–Gs
UV280 absorbance (mAU)
Volume (mL)
GLP1R-LgBit
DNGαs
Gβ1-HiBit
Nb35
Gγ2
kDa
d
Compound 2–GLP-1–GLP-1R–Gs
e
Compound 2–LY3502970–GLP-1R–Gs
miniGαs

Supplementary Fig. 1 Functional validation of the receptor constructs and purification of the complexes. **a**, Schematic diagram of the receptor constructs used for structure determination. **b**, GLP-1, compound 2 and LY3502970 induced cAMP accumulation. Data are shown as means ± S.E.M from three independent experiments performed in technical triplicate. **c-e**, Analytical size-exclusion chromatography, SDS-PAGE/Coomassie blue stain and representative negative staining image of the purified complexes: compound 2–GLP-1R–G_s complex (**c**), compound 2–GLP-1–GLP-1R–G_s complex (**d**) and compound 2–LY3502970–GLP-1R–G_s complex (**e**). These experiments were repeated independently twice with similar results. WT, wild-type. Source data are provided as a Source Data file.

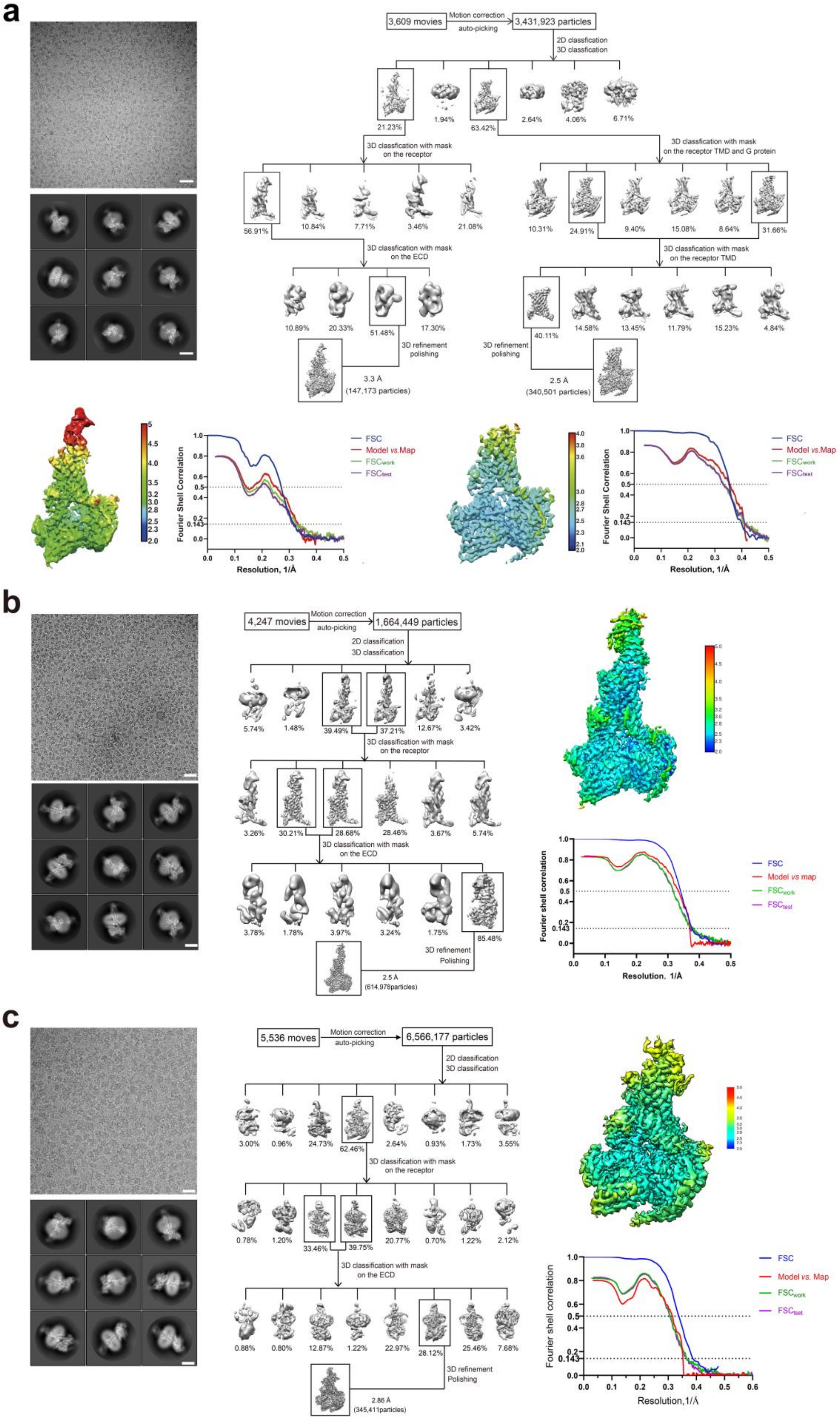
a
3,609 movies
Motion correction
auto-picking
3,431,923 particles
2D classfication
3D classfication
21.23%
1.94%
63.42%
2.64%
4.06%
6.71%
3D classfication with mask
on the receptor
3D classfication with mask
on the receptor TMD and G protein
56.91%
10.84%
7.71%
3.46%
21.08%
10.31%
24.91%
9.40%
15.08%
8.64%
31.66%
3D classfication with mask
on the ECD
3D classfication with mask
on the receptor TMD
10.89%
20.33%
51.48%
17.30%
40.11%
14.58%
13.45%
11.79%
15.23%
4.84%
3D refinement
polishing
3.3 Å
(147,173 particles)
2.5 Å
(340,501 particles)
FSC
Model vs.Map
FSCwork
FSCtest
Fourier Shell Correlation
Resolution, 1/Å
b
4,247 movies
Motion correction
auto-picking
1,664,449 particles
2D classification
3D classification
5.74%
1.48%
39.49%
37.21%
12.67%
3.42%
3D classification with mask
on the receptor
3.26%
30.21%
28.68%
28.46%
3.67%
5.74%
3D classification with mask
on the ECD
3.78%
1.78%
3.97%
3.24%
1.75%
85.48%
3D refinement
Polishing
2.5 Å
(614,978particles)
FSC
Model vs map
FSCwork
FSCtest
Fourier shell correlation
Resolution, 1/Å
c
5,536 moves
Motion correction
auto-picking
6,566,177 particles
2D classification
3D classification
3.00%
0.96%
24.73%
62.46%
2.64%
0.93%
1.73%
3.55%
3D classification with mask
on the receptor
0.78%
1.20%
33.46%
39.75%
20.77%
0.70%
1.22%
2.12%
3D classification with mask
on the ECD
0.88%
0.80%
12.87%
1.22%
22.97%
28.12%
25.46%
7.68%
3D refinement
Polishing
2.86 Å
(345,411particles)
FSC
Model vs. Map
FSCwork
FSCtest
Fourier shell correlation
Resolution,1/Å

Supplementary Fig. 2 Cryo-EM data processing and validation. **a**, Compound 2–GLP-1R–G_s complex: top left, representative cryo-EM micrograph (scale bar: 40 nm) and two-dimensional class averages (scale bar: 5 nm); top right, flow chart of cryo-EM data processing; bottom left, local resolution distribution map of the complex with the ECD and Gold-standard Fourier shell correlation (FSC) curves of overall refined receptor; bottom right, local resolution distribution map of the complex without the ECD and FSC curves of overall refined receptor. **b**, Compound 2–GLP-1–GLP-1R–G_s complex: left, representative cryo-EM micrograph (scale bar: 40 nm) and two-dimensional class averages (scale bar: 5 nm); middle, flow chart of cryo-EM data processing; right, local resolution distribution map of the complex and FSC curves of overall refined receptor. **c**, Compound 2–LY3502970–GLP-1R–G_s complex: left, representative cryo-EM micrograph (scale bar: 20 nm) and two-dimensional class averages (scale bar: 5 nm); middle, flow chart of cryo-EM data processing; right, local resolution distribution map of the complex and FSC curves of overall refined receptor. These experiments were repeated independently twice with similar results.

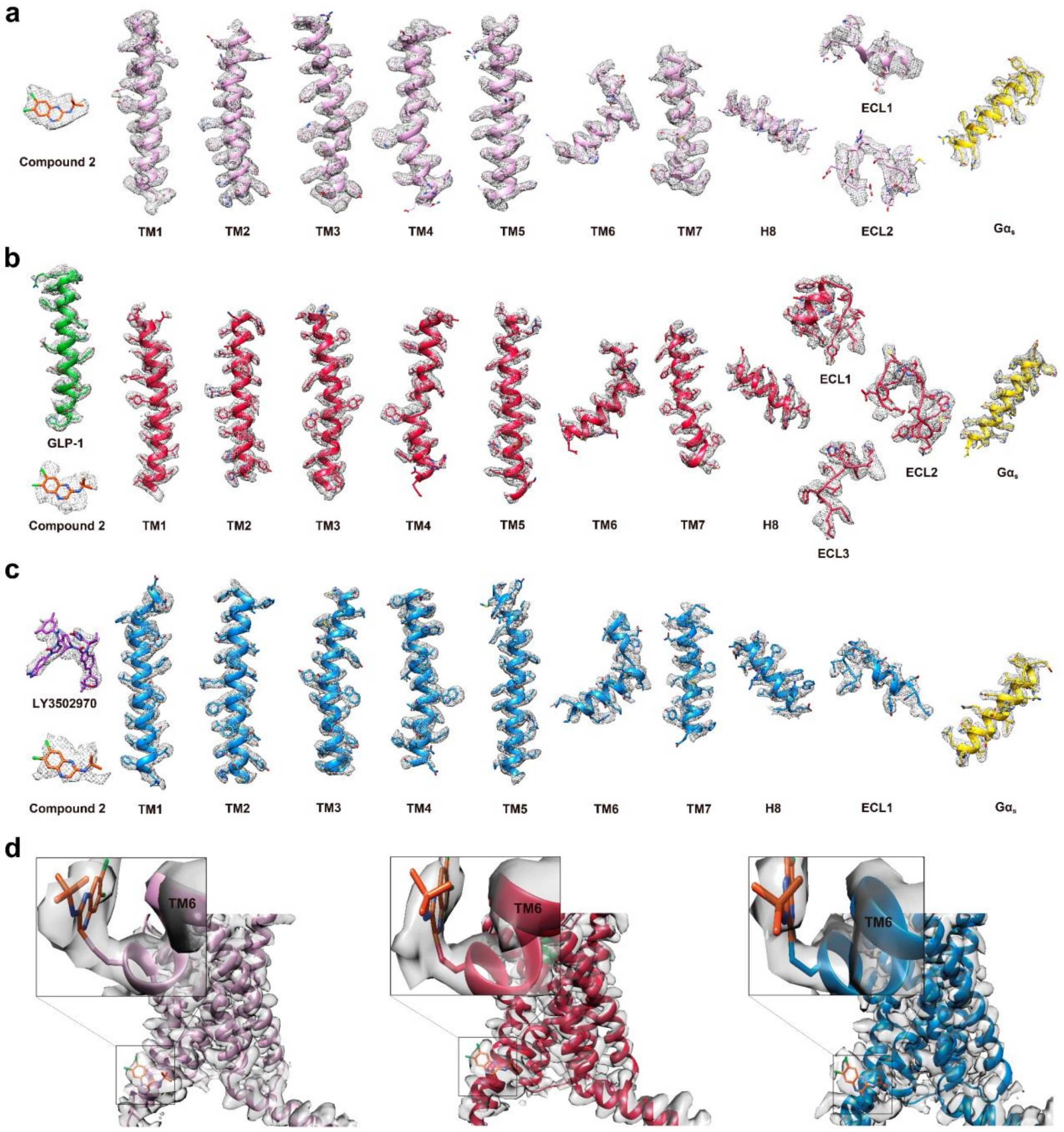

Supplementary Fig. 3 Near-atomic resolution model of the complexes in the cryo-EM density maps. a, EM density map and model of the compound 2–GLP-1R–G_s complex are shown for all seven-transmembrane (7TM) α-helices, helix 8 and all extracellular loops of GLP-1R, compound 2 and the α5-helix of the $Gα_s$ Ras-like domain. **b**, EM density map and model of the compound 2–GLP-1–GLP-1R–G_s complex are shown for all 7TM α-helices, helix 8 and all extracellular loops of GLP-1R, GLP-1, compound 2 and the α5-helix of the $Gα_s$ Ras-like domain. **c**, EM density map and model of the compound 2–LY3502970–GLP-1R–G_s complex are shown for all 7TM α-helices, helix 8 and ECL1 of GLP-1R, compound 2, LY3502970 and the α5-helix of the $Gα_s$ Ras-like domain. **d**, The zoomed-in views of compound 2 and the interacting region of TM6 placed inside the density maps of compound 2–GLP-1 (left), compound 2–GLP-1–GLP-1R (middle) and compound 2–LY3502970–GLP-1R (right).

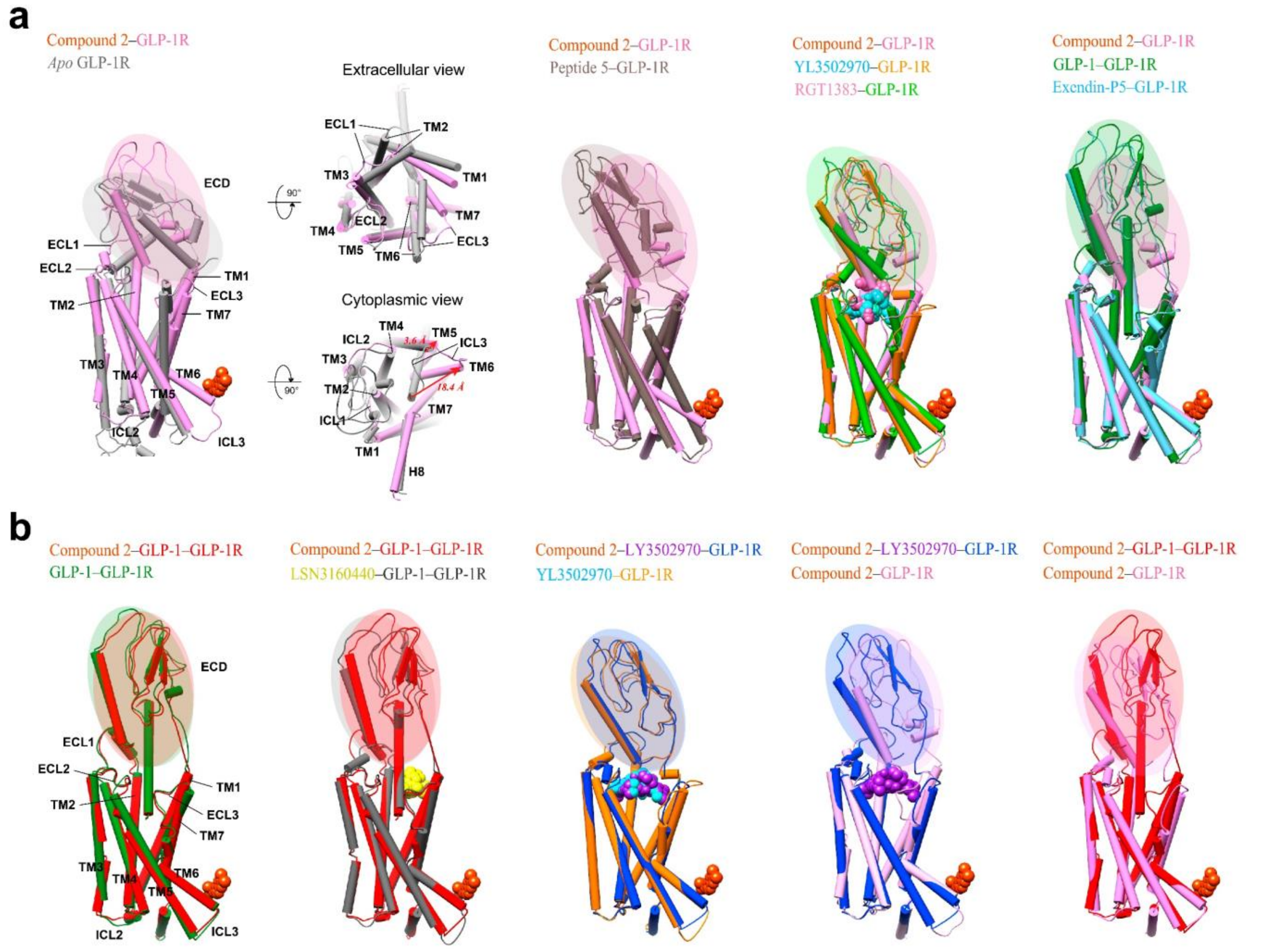

Supplementary Fig. 4 Comparison of available GLP-1R structures. a, Overlay of compound 2-bound GLP-1R with inactive, intermediate, peptide-bound and small molecule agonist-bound GLP-1R shows the agonism of compound 2. **b**, Overlay of compound 2 and GLP-1 or LY3502970-bound GLP-1R with related agonist bound GLP-1R shows the allosterism of compound 2. Colors of the GLP-1R and ligands are in line with the text above the structure.

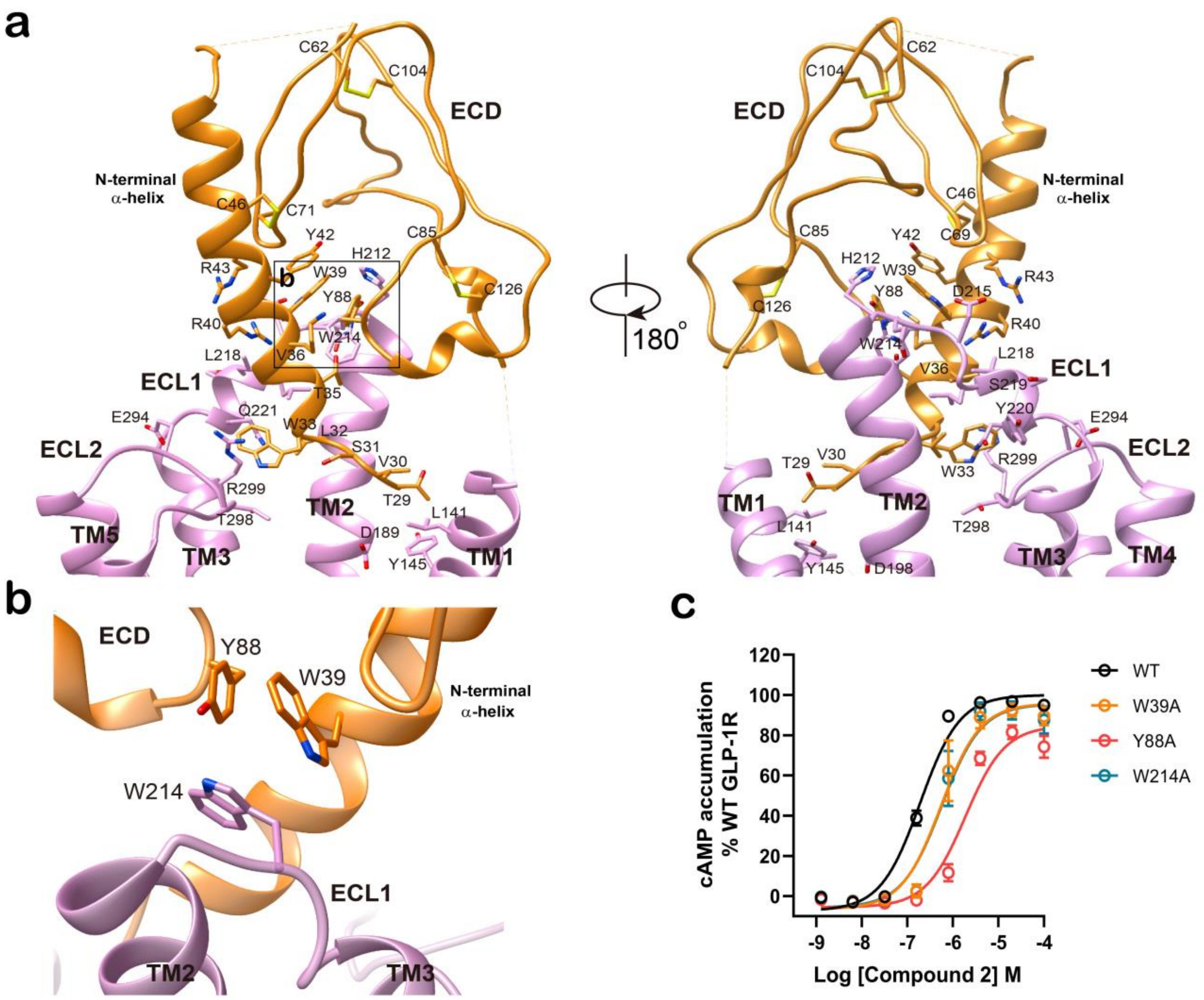

Supplementary Fig. 5 Unique ECD conformation in the compound 2-bound GLP-1R. a, The TMD-interacting conformation of the ECD. The ECD of GLP-1R (orange) folded down towards the TMD core and penetrated into the orthosteric binding pocket through its N-terminal α-helix. **b**, The ECD-ECL1 interactions. The ECD orientation is stabilized by interactions with ECL1. Important residues are shown in sticks. **c**, Effects of W39A, Y88A, and W214A mutants on compound 2-induced cAMP responses. Data shown are means ± S.E.M. of three independent experiments. WT, wild-type. Source data are provided as a Source Data file.

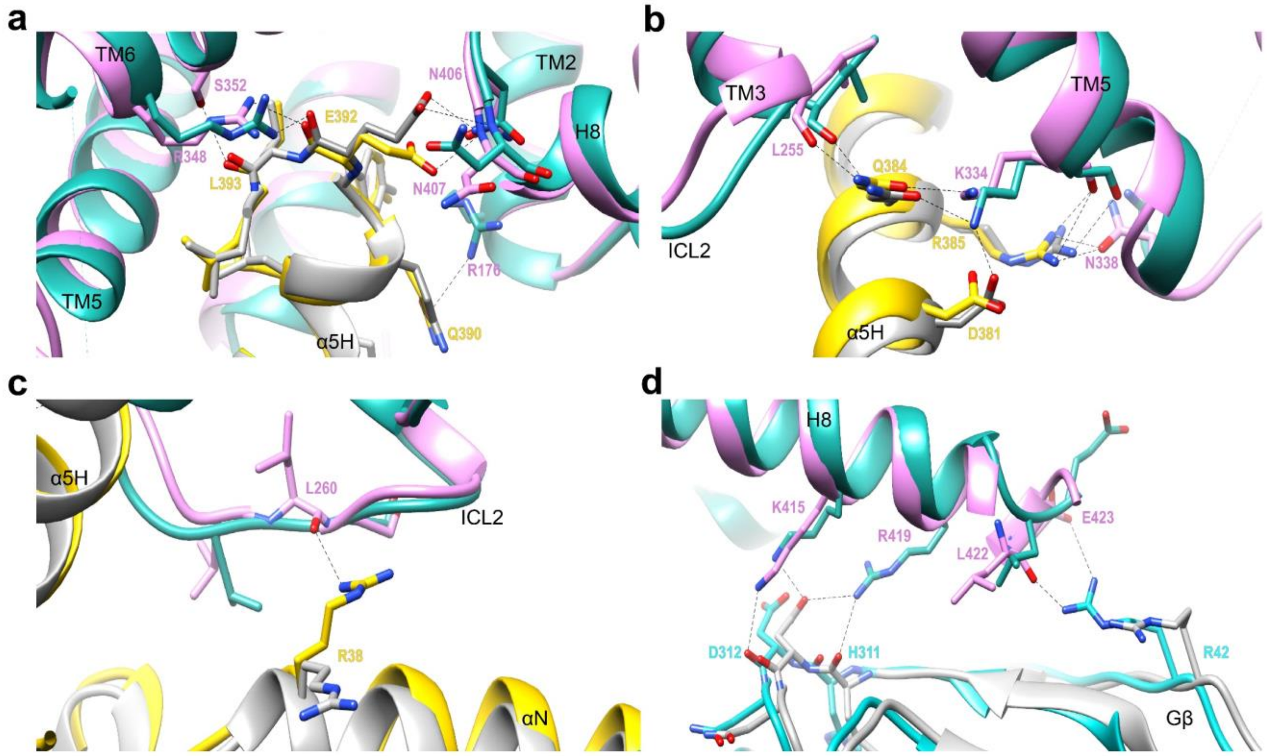

Supplementary Fig. 6 Comparison of G protein coupling between compound 2-bound and GLP-1-bound active GLP-1R in complex with G_s. a-b, GLP-1R–Gα_s α5 helix (α5H) interface. **c**, GLP-1R–Gα_s N-terminal helix (αN) interface. **D**, GLP-1R helix 8-Gβ interface. Compound 2-bound GLP-1R in hot pink; GLP-1-bound GLP-1R in sea green; Gαs Ras-like domain of compound 2–GLP-1R–G_s complex in yellow; Gβ subunit of compound 2–GLP-1R–G_s complex in cyan; Gαs Ras-like domain and Gβ subunit of GLP-1–GLP-1R–G_s complex (PDB: 6X18) in gray.

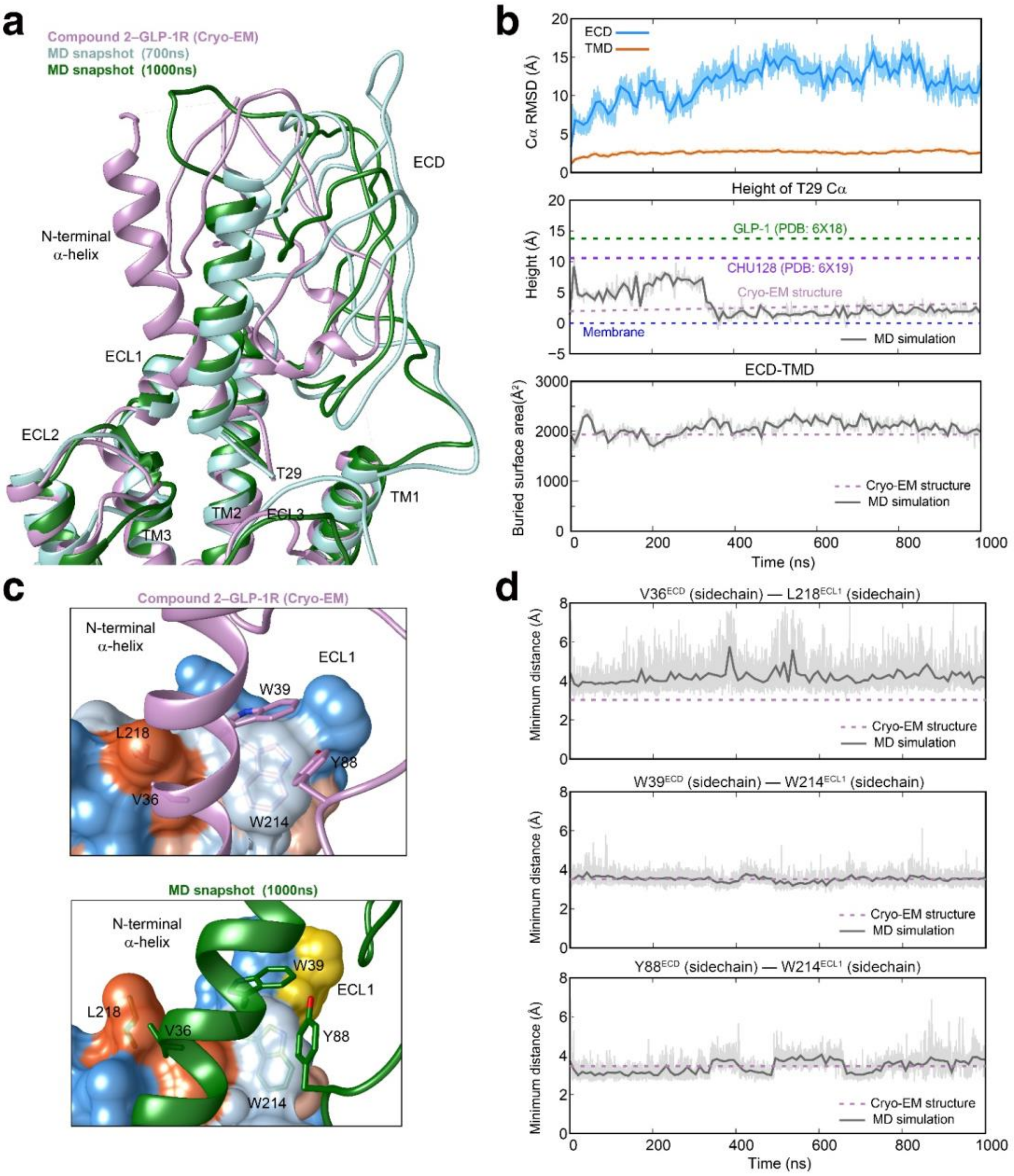

Supplementary Fig. 7 Molecular dynamics (MD) simulations of compound 2-bound active GLP-1R. a, Comparison of the ECD conformations between simulation snapshots and the cryo-EM structure (hot pink). **b**, Movements of the ECD during MD simulations: top, root mean square deviation (RMSD) of Cα positions of the GLP-1R, where all MD snapshots were superimposed on the cryo-EM structure of GLP-1R TMD using the Cα atoms; middle, the height of T29 Cα atom of the GLP-1R ECD relative to the membrane layer; bottom, the buried surface area between GLP-1R ECD and TMD. Interface areas were calculated using freeSASA. During the MD simulation, the N-terminal α-helix of the GLP-1R ECD consistently inserted to the TMD core, in line with the cryo-EM structure, evidenced by the height of its tip (T29) and the ECD-TMD buried surface area. The thick and thin traces represent moving averages and original, unsmoothed values, respectively. **c**, Contacts between ECD and ECL1 for the cryo-EM structure and MD snapshot. The ECL1 is shown in surface representation and colored in dodger blue for the most hydrophilic region and orange red for the most hydrophobic region, respectively. **d**, Three distances between side chain heavy atoms from the residues on the ECD and ECL1 (Top, $V36^{ECD}-L218^{ECL1}$; middle, $W39^{ECD}-W214^{ECL1}$; bottom, $Y88^{ECD}-W214^{ECL1}$). The thick and thin traces represent moving averages and original, unsmoothed values, respectively.

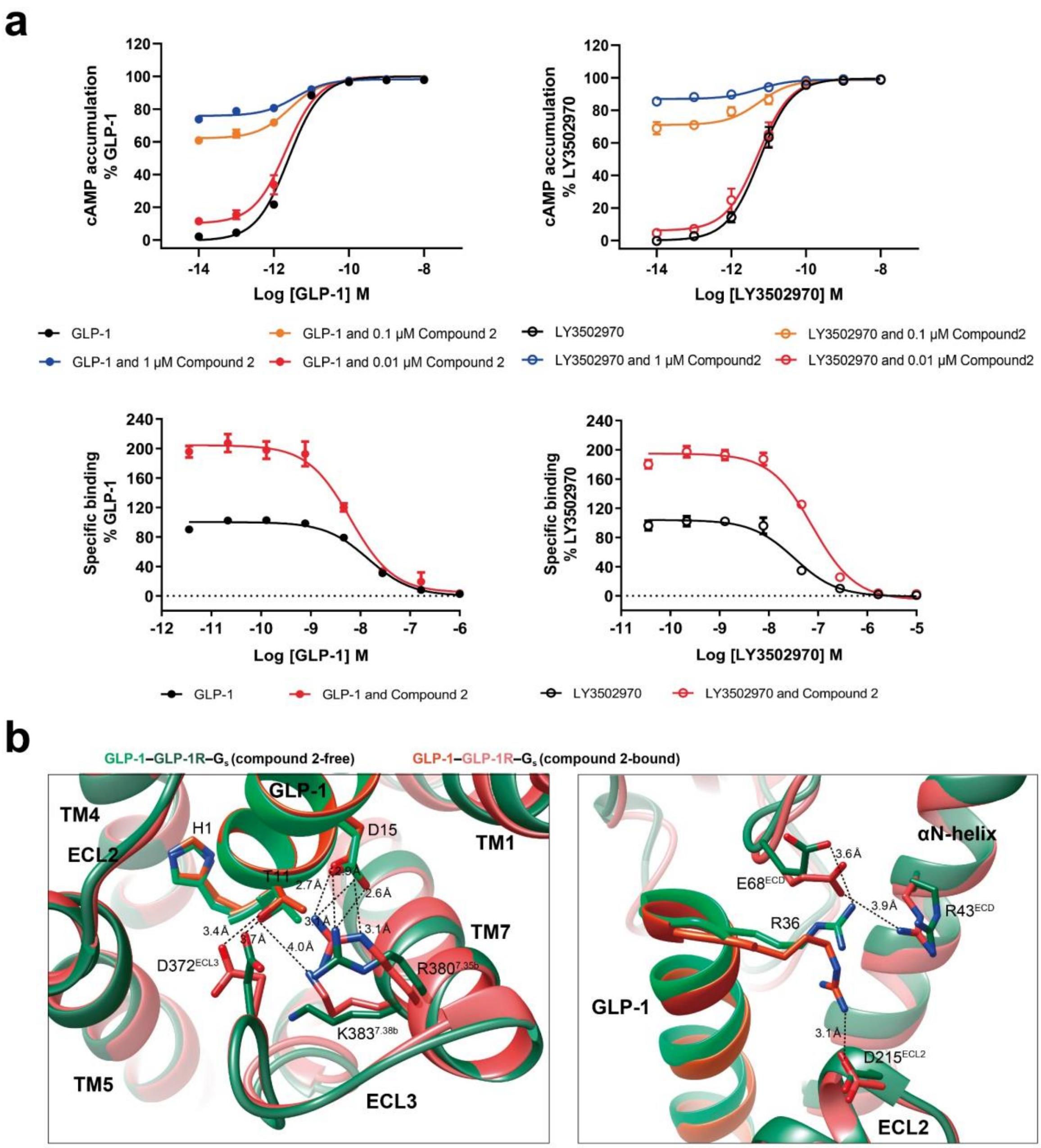

Supplementary Fig. 8 Potentiation of GLP-1 and LY3502970 activity by compound 2. a, Effects of compound 2 on cAMP signaling and GLP-1R binding: top, dose–response characteristics of GLP-1 and LY3502970 in the absence or presence of compound 2 at three different concentrations. In line with previous findings[24], we did not find any increase of GLP-1 potency following of compound 2 treatment (EC_{50} was 2.48, 3.63, 2.63 and 2.13 pM for GLP-1 in the presence of 0, 0.1, 0.01, and 1 μM compound 2, respectively), suggesting that compound 2 only potentiates GLP-1-induced receptor activation. The same phenomenon was observed for LY3502970 (EC_{50} was 5.67, 5.31, 5.51 and 5.17 pM for LY3502970 in the presence of 0, 0.1, 0.01, and 1 μM compound 2, respectively); bottom, competitive binding of ^{125}I-labelled GLP-1 with GLP-1 or LY3502970 at GLP-1R in the presence of compound 2. The results show that compound 2 enhances GLP-1 and LY3502970 binding with GLP-1R by 2-fold and 1.9-fold, respectively. Data shown are means ± S.E.M. from at least three independent experiments (n=4-6) performed in duplicate (receptor binding assay) or quadruplicate (cAMP accumulation). Source data are provided as a Source Data file. **b**, Comparison of GLP-1 and GLP-1R interaction in the presence and absence of compound 2. The cryo-EM structure of GLP-1–GLP-1R–G_s (PDB code: 6X18) was superimposed on Cα atoms of the compound 2–GLP-1–GLP-1R–G_s complex.

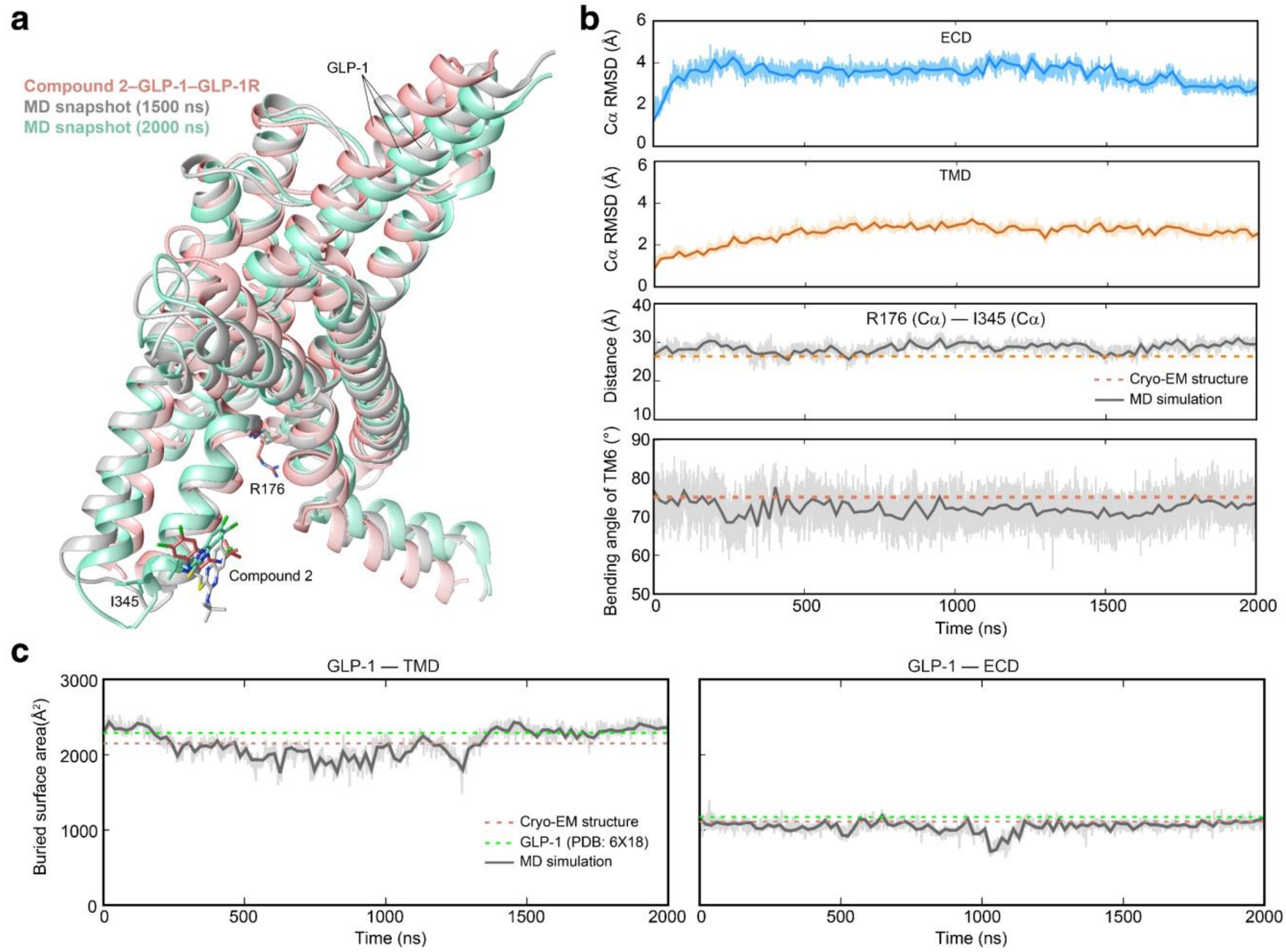

Supplementary Fig. 9 MD simulations of compound 2–GLP-1–GLP-1R. a, Compassion of receptor conformations between simulation snapshots and the cryo-EM structure (hot pink). ECD (residues 29 to 136) and G protein are omitted for clarity. **b**, Conformational movements of GLP-1R simulations: top, root mean square deviation (RMSD) of Cα positions of the GLP-1R ECD, which were superimposed on the ECD of the cryo-EM structure using the Cα atoms; upper middle, RMSD of Cα positions of the GLP-1R TMD (residues 137 to 423), which were superimposed on the TMD of the cryo-EM structure using the Cα atoms; lower middle, the Cα distance between two intracellular residues (R176 and I345); bottom, bending angle of TM6 (measured by the angle from L356$^{6.45b}$ Cα to T362$^{6.51b}$ Cα via L359$^{6.48b}$ Cα). The thick and thin traces represent moving averages and original, unsmoothed values, respectively. **c**, The buried surface area between GLP-1 and GLP-1R. Interface areas were calculated using freeSASA. The thick and thin traces represent moving averages and original, unsmoothed values, respectively.

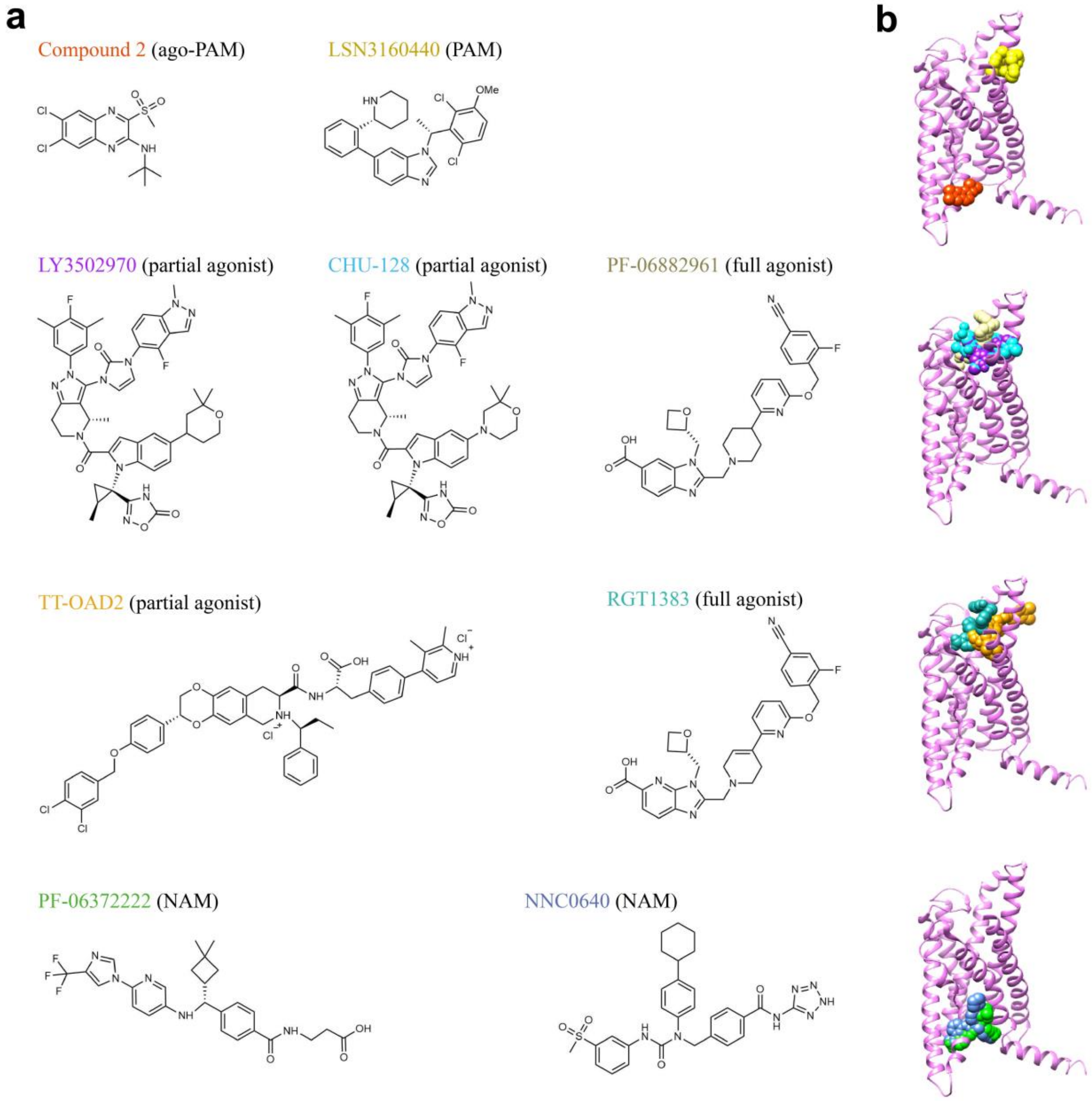

Supplementary Fig. 10 List of small molecule GLP-1R modulators with available structures. **a**, Chemical structures of small molecule ligands. **b**, Binding sites of the small molecules relative to the compound 2–GLP-1R–G_s complex (magenta). PAM, positive allosteric modulator; NAM, negative allosteric modulator.

Supplementary Table. 1 Cryo-EM data collection, refinement and validation statistics.

	Compound 2-GLP-1R-G_s (without ECD)	Compound 2-GLP-1R-G_s (with ECD)	Compound 2-GLP-1-GLP-1R-G_s	Compound 2-LY3502970-GLP-1R-G_s
**Data collection and processing**				
Magnification	81000	81000	81000	81000
Voltage (kV)	300	300	300	300
Electron exposure (e–/Å^2)	80	80	80	80
Defocus range (μm)	-1.2 to -2.2	-1.2 to -2.2	-1.2 to -2.2	-1.2 to -2.2
Pixel size (Å)	1.045	1.045	1.045	1.045
Symmetry imposed	C1	C1	C1	C1
Initial particle images (no.)	502,346	1,500,650	1, 664,449	65,661,77
Final particle images (no.)	147,173	340,350	614978	345411
Map resolution (Å)	3.3	2.5	2.5	2.9
FSC threshold	0.143	0.143	0.143	0.143
Map resolution range (Å)	2.8-5.0	2.3-5.0	2.2-5.0	2.7-5.0
Map sharpening *B* factor (Å^2)	-72	-77	-77	-98
**Refinement**				
Initial model used (PDB code)	6VCB	6VCB	6X18	6B3J
Model resolution (Å)	2.9	2.9	2.8	2.9
FSC threshold	0.5	0.5	0.5	0.5
Model resolution range (Å)	2.3-5.0	2.3-5.0	2.2-5.0	2.7-5.0
Model composition				
Non-hydrogen atoms	8,583	9,229	9,612	8,802
Protein residues	1,040	1,128	1,174	1,089
Water	0	0	0	0
B factors (Å^2)				
Protein	99.07	133.33	31.03	38.41
Ligand	128.62	99.13	39.12	36.76
R.m.s. deviations				
Bond lengths (Å)	0.008	0.006	0.006	0.002
Bond angles (°)	0.687	0.941	0.729	0.550
Validation				
MolProbity score	1.75	2.44	1.62	1.47
Clashscore	8.27	13.44	10.68	7.26
Poor rotamers (%)	0.22	0.19	0.20	0.11
Ramachandran plot				
Favored (%)	95.61	95.94	97.66	97.65
Allowed (%)	4.30	4.81	2.34	2.35
Disallowed (%)	0.00	0.09	0.00	0.00
Real space correlation coefficient				
Compound 2	0.59	0.54	0.47	0.46
LY3502970				0.74

Supplementary Table. 2 *In vitro* pharmacology of GLP-1R.

	cAMP accumulation		[125I] GLP-1 binding
	**GLP-1**		
	pEC_{50}	E_{max}	pKi
WT GLP-1R	11.19±0.07	98.6±1.96	8.51±0.07
GLP-1R-LgBiT-2MBP	11.24±0.09	97.64±2.53	8.71±0.24
Δ28	9.53±0.15**	102.8±7.30	NB**
Δ33	10.17±0.139**	98.2±5.47	NB**
Δ38	NA**	NA**	NB**
Δ43	NA**	NA**	NB**
Δ48	NA**	NA**	NB**
Δ55	NA**	NA**	NB**
ΔECD	8.89±0.66**	6.88±2.85**	NB**
V332A	10.85±0.23	109.4±2.53*	8.87±0.13*
C347A	10.58±0.17	102.1±5.53	8.81±0.11
K346A	10.88±0.4	100.2±3.36	8.89±0.12*
L349A	10.53±0.26*	107.1±5.01	9.41±0.11**
A350W	9.55±0.1**	99.17±3.31	9.40±0.12**
K351A	10.06±0.12**	108.6±4.61*	8.40±0.24
	**Compound 2**[a]		
WT GLP-1R	6.13±0.06	99.7±2.27	
GLP-1R-LgBiT-2MBP	5.99±0.08	98.4±2.97	
Δ28	6.19±0.17	67.94±3.78**	
Δ33	6.03±0.16	80.04±4.81**	
Δ38	6.20±0.15	61.38±3.00**	
Δ43	6.12±0.19	70.27±4.57**	
Δ48	6.24±0.28	62.19±5.49**	
Δ55	7.01±2.33	10.79±0.60**	
ΔECD	6.11±0.82	2.22±2.73**	
V332A	6.58±0.31	104.7±2.53	
C347A	6.0±0.6	7.67±0.84**	
K346A	6.55±0.24	102.5±1.73	
L349A	6.37±0.22	101.4±3.14	
A350W	5.47±0.96	29.28±6.6**	
K351A	5.83±2.15	20.11±6.27**	
	**LY3502970**		
WT GLP-1R	10.65±0.05	99.89±1.72	7.77±0.10
GLP-1R-LgBiT-2MBP	11.09±0.06	98.07±1.94	7.80±0.24

Binding data were analyzed using a three-parameter logistic equation and normalized to the maximal binding of [125I] GLP-1. pKi is the negative logarithm of peptide affinity. cAMP accumulation data were analyzed using a three-parameter logistic equation to determine pEC_{50} and E_{max} values. pEC_{50} is the negative logarithm of the molar concentration of agonist that induced half the maximal response. E_{max} for mutants is expressed as a percentage of the wild-type (WT). All values are means±S.E.M of at least three independent experiments conducted in duplicate. One-way ANOVA was used to determine statistical significance (**P<0.01, *P<0.05). NA, not active; insufficient

radiolabeled ligand displacement was detected implying GLP-1 binding impairment; Δ, residue truncation. Source data are provided as a Source Data file. [a]According to previously report[24], compound 2 does not compete radiolabeled GLP-1 binding to GLP-1R and hence, it was not assessed for receptor binding.

Supplementary Table. 3 *In vitro* pharmacology of GCGR.

	cAMP accumulation	
	**Glucagon**	
	pEC_{50}	E_{max}
WT GCGR	8.48±0.08	99.39±3.68
F345C	8.43±0.08	95.36±3.70
F345C Δ30	9.06±0.89**	7.93±1.69**
F345C Δ35	8.14±0.57	6.9±3.27**
F345C Δ40	8.91±0.39*	6.66±1.11**
F345C Δ45	8.28±0.45	4.35±1.88**
F345C Δ50	9.05±0.65	3.15±1.53**
F345C Δ55	8.70±0.89	-1.93±2.79**
	**Compound 2**	
WT GCGR	6.74±0.83	11.89±1.01**
F345C	6.04±0.08	99.87±3.16
F345C Δ30	5.80±0.15	64.39±3.92**
F345C Δ35	5.88±0.20	52.0±4.0**
F345C Δ40	6.09±0.49	26.97±3.47**
F345C Δ45	5.86±0.44	28.43±3.73**
F345C Δ50	6.37±0.67	14.24±2.28**
F345C Δ55	6.03±0.45	24.37±3.48**

cAMP accumulation data were analyzed using a three-parameter logistic equation to determine pEC_{50} and E_{max} values. pEC_{50} is the negative logarithm of the molar concentration of agonist that induced half the maximal response. E_{max} for mutants is expressed as a percentage of the wild-type (WT). All values are means ± S.E.M of at least three independent experiments conducted in duplicate. One-way ANOVA was used to determine statistical significance (**P<0.01, *P<0.05). Δ, residue truncation. Source data are provided as a Source Data file.

Supplementary Table. 4 Effects of residue mutation in the ECD-ECL1 interface on cAMP accumulation.

	Compound 2	
	pEC_{50}	E_{max}
WT GLP-1R	6.72±0.05	99.99±1.93
W39A	6.26±0.12**	95.79±4.51
Y88A	5.72±0.09**	84.74±4.06*
W214A	6.24±0.11**	95.54±3.96

cAMP accumulation data were analyzed using a three-parameter logistic equation to determine pEC_{50} and E_{max} values. pEC_{50} is the negative logarithm of the molar concentration of agonist that induced half the maximal response. E_{max} for mutants is expressed as a percentage of the wild-type (WT). All values are means ± S.E.M of at least three independent experiments conducted in duplicate. One-way ANOVA was used to determine statistical significance (**$P<0.01$, *$P<0.05$). Source data are provided as a Source Data file.

Supplementary Table. 5 Details of restraints applied during MD simulations.

Stage	Time step	Simulation time	Restrain
Heating	1 fs	1 ns	Position harmonic restrain (40 $kJ\cdot mol^{-1}\cdot Å^{-2}$) for the backbone non-hydrogen atoms of protein and peptide;<br>Position restrain (20 $kJ\cdot mol^{-1}\cdot Å^{-2}$) for the sidechain non-hydrogen atoms of protein and peptide;<br>Planar harmonic restraint (10 $kJ\cdot mol^{-1}\cdot Å^{-2}$) for the phosphorus atom of POPC along the Z-axis;<br>Dihedral restraint (1000 $kJ\cdot mol^{-1}\cdot rad^{-2}$) for two dihedrals (C28-C29-C210-C211 and C1-C3-C2-O21).
Step6.1	1 fs	5 ns	Position harmonic restrain (40 $kJ\cdot mol^{-1}\cdot Å^{-2}$) for the backbone non-hydrogen atoms of protein and peptide;<br>Position restrain (20 $kJ\cdot mol^{-1}\cdot Å^{-2}$) for the sidechain non-hydrogen atoms of protein and peptide;<br>Planar harmonic restraint (10 $kJ\cdot mol^{-1}\cdot Å^{-2}$) for the phosphorus atom of POPC along the Z-axis;<br>Dihedral restraint (1000 $kJ\cdot mol^{-1}\cdot rad^{-2}$) for two dihedrals (C28-C29-C210-C211 and C1-C3-C2-O21).
Step6.2	1 fs	5 ns	Position harmonic restrain (20 $kJ\cdot mol^{-1}\cdot Å^{-2}$) for the backbone non-hydrogen atoms of protein and peptide;<br>Position restrain (10 $kJ\cdot mol^{-1}\cdot Å^{-2}$) for the sidechain non-hydrogen atoms of protein and peptide;<br>Planar harmonic restraint (4 $kJ\cdot mol^{-1}\cdot Å^{-2}$) for the phosphorus atom of POPC along the Z-axis;<br>Dihedral restraint (400 $kJ\cdot mol^{-1}\cdot rad^{-2}$) for two dihedrals (C28-C29-C210-C211 and C1-C3-C2-O21).
Step6.3	1 fs	10 ns	Position harmonic restrain (10 $kJ\cdot mol^{-1}\cdot Å^{-2}$) for the backbone non-hydrogen atoms of protein and peptide;<br>Position restrain (5 $kJ\cdot mol^{-1}\cdot Å^{-2}$) for the sidechain non-hydrogen atoms of protein and peptide;<br>Planar harmonic restraint (4 $kJ\cdot mol^{-1}\cdot Å^{-2}$) for the phosphorus atom of POPC along the Z-axis;<br>Dihedral restraint (200 $kJ\cdot mol^{-1}\cdot rad^{-2}$) for two dihedrals (C28-C29-C210-C211 and C1-C3-C2-O21).
Step6.4	1 fs	10 ns	Position harmonic restrain (5 $kJ\cdot mol^{-1}\cdot Å^{-2}$) for the backbone non-hydrogen atoms of protein and peptide;<br>Position restrain (2 $kJ\cdot mol^{-1}\cdot Å^{-2}$) for the sidechain non-hydrogen atoms of protein and peptide;<br>Planar harmonic restraint (2 $kJ\cdot mol^{-1}\cdot Å^{-2}$) for the phosphorus atom of POPC along the Z-axis;<br>Dihedral restraint (200 $kJ\cdot mol^{-1}\cdot rad^{-2}$) for two dihedrals (C28-C29-C210-C211 and C1-C3-C2-O21).
Step6.5	1 fs	10 ns	Position harmonic restrain (2 $kJ\cdot mol^{-1}\cdot Å^{-2}$) for the backbone non-hydrogen atoms of protein and peptide;<br>Position restrain (0.5 $kJ\cdot mol^{-1}\cdot Å^{-2}$) for the sidechain non-hydrogen atoms of protein and peptide;<br>Planar harmonic restraint (0.4 $kJ\cdot mol^{-1}\cdot Å^{-2}$) for the phosphorus atom of POPC along the Z-axis;<br>Dihedral restraint (100 $kJ\cdot mol^{-1}\cdot rad^{-2}$) for two dihedrals (C28-C29-C210-C211 and C1-C3-C2-O21).
Step6.6	1 fs	10 ns	Position harmonic restrain (0.5 $kJ\cdot mol^{-1}\cdot Å^{-2}$) for the backbone non-hydrogen atoms of protein and peptide;
Step7	2fs	1000 ns	Restrain-free

Supplementary Table. 6 Primers used in this study.

Related data	Primers	Sequence (5'-3')
	**Primers of GLP-1R mutants**	
cAMP assay in Supplementary Fig. 5	W39A-Forward	GCAGAAAGCCCGAGAATACCGACGCCAGTGCC
	W39A-Reverse	ATTCTCGGCCTTTCTGCACCGTCTCCCAGAGG
	Y88A-Forward	AGCTGCCCCTGGGCCCTGCCCTGGGCCAGCAGT
	Y88A-Reverse	AGGGCCCAGGGGCAGCTGACATTCACGAACGA
	W214A-Forward	AGCAGCACCAGGCCGATGGGCTCCTCTCCTACCAG
	W214A-Reverse	ATCGGCCTGGTGCTGCTGGGCGGCTGTGCTAT
cAMP assay in Fig. 2c	V332A-Forward	ATCGTGGCCTCCAAACTGAAGGCCAATCTCAT
	V332A-Reverse	AGTTTGGAGGCCACGATGCAGATGACCCGAAC
	K346A-Forward	ACATCGCCTGCAGACTTGCCAAGTCCACGCTG
	K346A-Reverse	AAGTCTGCAGGCGATGTCTGTCTTGCACATGAGATTG
	C347A-Forward	GAGATTGGCCAGACTTGCCAAGTCCACGCTGA
	C347A- Reverse	CAAGTCTGGCCAATCTCATGTGCAAGACAGACATC
	L349A-Forward	AAATGCAGAGCCGCCAAGTCCACGCTGACACT
	L349A-Reverse	TTGGCGGCTCTGCATTTGATGTCTGTCTTGCA
	A350W-Forward	CAGACTTTGGAAGTCCACGCTGACACTCATCC
	A350W-Reverse	TGGACTTCCAAAGTCTGCATTTGATGTCTGTCTTG
	K351A-Forward	ATGCAGACTTGCCGCCTCCACGCTGACACTCATCCC
	K351A-Reverse	AGGCGGCAAGTCTGCATTTGATGTCTGTCTTG
cAMP assay in Fig. 3d	Δ28-Forward	TGGTATTCGCCACTGTGTCCCTCTGGGAGACGG
	Δ28-Reverse	ACACAGTGGCGAATACCAGGCAGAAGATGTAGC
	Δ33-Forward	CCGAGACGGTGCAGAAATGGCGAGAATACCGAC
	Δ33-Reverse	ATTTCTGCACCGTCTCGGCGAATACCAGGCAGAAGA
	Δ38-Forward	TATTCGCCTGGCGAGAATACCGACGCCAGTGCC
	Δ38-Reverse	ATTCTCGCCAGGCGAATACCAGGCAGAAGATGT
	Δ43-Forward	TTCTGCCTGGTATTCGCCCGCCAGTGCCAGCGCTCC
	Δ43-Reverse	GGCGAATACCAGGCAGAAGATGTAGCTCAGGGC
	Δ48-Forward	GTATTCGCCTCCCTGACTGAGGATCCACCTCCT
	Δ48-Reverse	AGTCAGGGAGGCGAATACCAGGCAGAAGATGTA
	Δ55-Forward	TTCTGCCTGGTATTCGCCCCACCTCCTGCCACAGACTTG
	Δ55-Reverse	GGCGAATACCAGGCAGAAGATGTAGCTCAGGGC
	**Primers of GCGR mutants**	
cAMP assay in Fig. 3d	F345C-Forward	ACAGACTACAAGTGCCGGCTGGCCAAGTCCACG
	F345C-Reverse	CGGCACTTGTAGTCTGTGTGGTGCATCTGCCG
	Δ30/F345C-Forward	CCTCCTTCCTGTTTGAGAAGTGGAAGCTCTACG
	Δ30/F345C-Reverse	TCTCAAACAGGAAGGAGGGGACCTGTGGCTGGC
	Δ35/F345C-Forward	TCTCAAACAGGAAGGAGGGGACCTGTGGCTGGC
	Δ35/F345C-Reverse	ACCGTAGAGCTTCCAGGAGGGGACCTGTGGCTG
	Δ40/F345C-Forward	CGACCAGTGTCACCACAACCTGAGCCTGCTGCC
	Δ40/F345C-Reverse	TTGTGGTGACACTGGTCGGAGGGGACCTGTGGCTG
	Δ45/F345C-Forward	TTGTGGTGACACTGGTCGGAGGGGACCTGTGGCTG

	Δ45/F345C-Reverse	TTGTGGTGACACTGGTCGGAGGGGACCTGTGGCTG
	Δ50//F345C-Forward	AGCCACAGGTCCCCTCCCCCCCTCCCACGGAGCTG
	Δ50//F345C-Reverse	GGGAGGGGACCTGTGGCTGGCAGGCCAGCAGCA
	Δ55/F345C-Forward	TCCCTGGTGTGCAACAGAACCTTCGACAAGTAT
	Δ55/F345C-Reverse	TCTGTTGCACACCAGGGAGGGGACCTGTGGCTG
